# Effects of the neonatal intensive care environment on circadian health and development of preterm infants

**DOI:** 10.3389/fphys.2023.1243162

**Published:** 2023-08-31

**Authors:** D. Van Gilst, A. V. Puchkina, J. A. Roelants, L. Kervezee, J. Dudink, I. K. M. Reiss, G. T. J. Van Der Horst, M. J. Vermeulen, I. Chaves

**Affiliations:** ^1^ Department of Molecular Genetics, Erasmus University Medical Center Rotterdam, Rotterdam, Netherlands; ^2^ Department of Developmental Biology, Erasmus University Medical Center Rotterdam, Rotterdam, Netherlands; ^3^ Department of Neonatal and Pediatric Intensive Care, Division of Neonatology, Erasmus University Medical Center Rotterdam-Sophia Children’s Hospital, Rotterdam, Netherlands; ^4^ Department of Cell and Chemical Biology, Leiden University Medical Center, Leiden, Netherlands; ^5^ Department of Neonatology, Wilhelmina Children’s Hospital, University Medical Center Utrecht, Utrecht, Netherlands

**Keywords:** circadian rhythm, development, NICU, chrono-nutrition, health, chronobiology, clock, cycled light field code changed

## Abstract

The circadian system in mammals ensures adaptation to the light-dark cycle on Earth and imposes 24-h rhythmicity on metabolic, physiological and behavioral processes. The central circadian pacemaker is located in the brain and is entrained by environmental signals called Zeitgebers. From here, neural, humoral and systemic signals drive rhythms in peripheral clocks in nearly every mammalian tissue. During pregnancy, disruption of the complex interplay between the mother’s rhythmic signals and the fetal developing circadian system can lead to long-term health consequences in the offspring. When an infant is born very preterm, it loses the temporal signals received from the mother prematurely and becomes totally dependent on 24/7 care in the Neonatal Intensive Care Unit (NICU), where day/night rhythmicity is usually blurred. In this literature review, we provide an overview of the fetal and neonatal development of the circadian system, and short-term consequences of disruption of this process as occurs in the NICU environment. Moreover, we provide a theoretical and molecular framework of how this disruption could lead to later-life disease. Finally, we discuss studies that aim to improve health outcomes after preterm birth by studying the effects of enhancing rhythmicity in light and noise exposure.

## Introduction

Throughout gestation, the developing fetus is fully taken care of within the uterine environment of its mother. Via the placenta, the mother sustains temperature control and provides oxygen, nutrients, and hormones. Inevitably, this means the fetus is exposed to the daily rhythms in maternal activity, food intake, and hormones such as melatonin and cortisol (Reiter et al., 2014a; Bates and Herzog, 2020). In case of very preterm birth (birth before 32 weeks of gestation), the neonate abruptly transitions from this controlled uterine environment into the chaotic reality of the neonatal intensive care unit (NICU). Over the past decades extensive guidelines on perinatal care have been developed, providing a framework for feeding schedules, temperature regulation and treatment of morbidities (American Academy of Pediatrics, Guidelines for perinatal care, 2017). A subject often overlooked within these guidelines is the implementation of rhythmic cues in clinical care, such as the light/dark cycle and feeding rhythms. Recently, Hazelhoff et al. (2021) discussed that cycled light in the NICU is beneficial for the alignment and development of the circadian system of the preterm infant. However, they did not shed light on the relevance of other environmental factors that may influence the circadian development of preterm infants in the NICU. The aim of this review is to provide an overview of the environmental factors, present in the NICU, that likely influence circadian entrainment of the preterm infant. Furthermore, we shine light on the underlying theoretical and molecular framework on how these conditions may program the preterm infant’s circadian system, and possibly affect their long-term health. Finally, we elaborate on attempts to enhance the rhythmicity of the NICU environment to improve short- and long-term development and health of preterm infants and discuss which circadian cues should additionally be taken into account.

## The circadian system

Circadian rhythms are generated by an internal circadian clock that allows adaptation of physiological and behavioral functions to the light-dark cycle on earth. It is coordinated by the central pacemaker, the paired suprachiasmatic nuclei (SCN), located bilaterally in the hypothalamus. Since the internal rhythm is not exactly 24 h, the circadian clock requires daily synchronization. The synchronization is mediated by light, which is the strongest Zeitgeber. A non-visual light signal is transmitted from a subset of retinal ganglion cells containing melanopsin photoreceptors to the central clock in the SCN. In addition to the SCN, there are peripheral clocks present in almost all other organs of the body, including the uterus and placenta (Reppert et al., 1988; Akiyama et al., 2010; Waddell et al., 2012). From the SCN, temporal information is transferred to peripheral circadian clocks via the autonomic nervous system and endocrine signals. Other factors such as food intake and physical activity or stress also contribute to synchronization of peripheral clocks (Husse et al., 2015; Do, 2019). In addition, evidence shows that peripheral clocks can be targeted directly by changes in their local environment such as temperature changes (Sumova et al., 2006). Signals received by the SCN or peripheral cells are processed and lead to synchronization of intracellular molecular clocks that impose 24 h rhythmicity on gene expression. Hence, this system ensures that the body can adapt its physiology to different phases of the day.

At the molecular level, oscillations are generated through transcriptional/translational feedback loops composed of clock genes and by posttranslational modifications ensuring rhythmic protein synthesis and degradation of clock proteins (Reppert et al., 1988). More specifically, the transcription factors CLOCK and BMAL1 heterodimerize and activate the transcription of Period (*PER1-3*) and Cryptochrome (*CRY1-2*) genes (Mendoza-Viveros et al., 2017). PER and CRY proteins, in turn, translocate into the nucleus where they inhibit the transcriptional activity of CLOCK:BMAL1 complexes, and thus their own synthesis (Mendoza-Viveros et al., 2017). Additionally, another feedback loop involving reverse erythroblastosis virus α (REV-ERBα) and retinoic acid receptor-related orphan receptor α (RORα) ensures stabilizing of this oscillation (Mendoza-Viveros et al., 2017). Together, these molecular mechanisms ensure a 24-h rhythm in most organs.

## Circadian development during pregnancy

**FIGURE 1:**
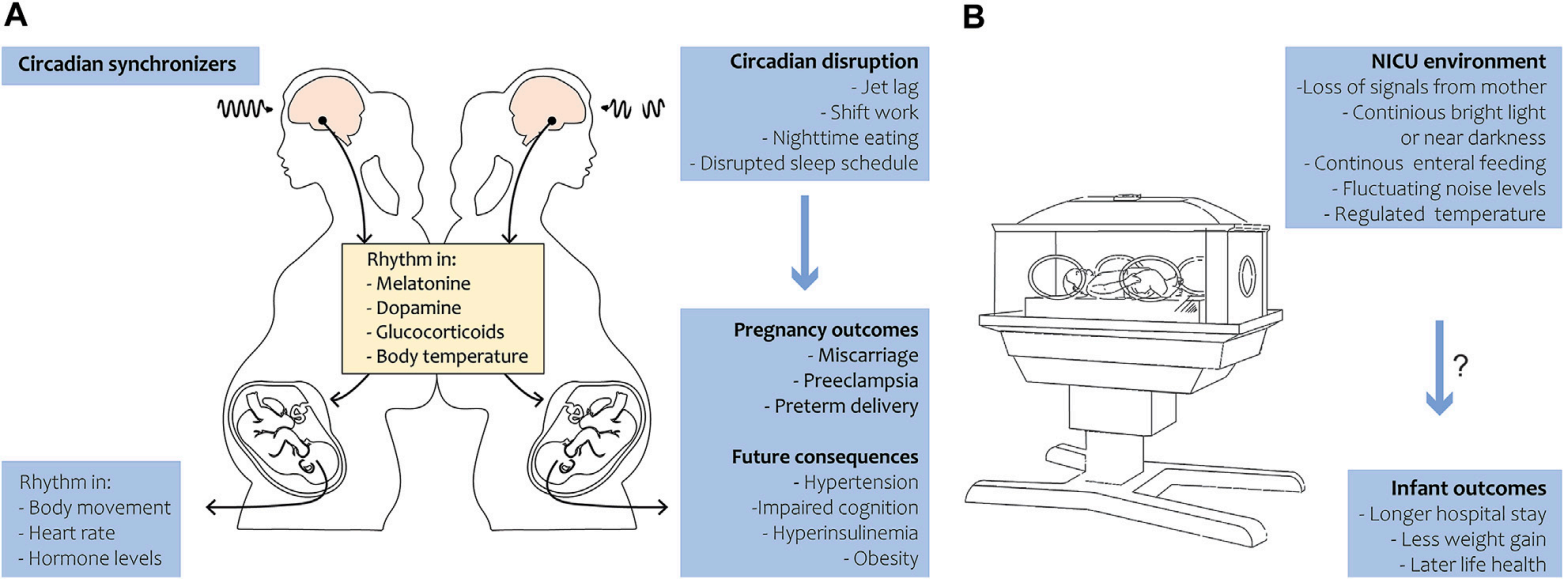
Circadian rhythm during pregnancy and in preterm birth. **(A)** Under the regulation of circadian synchronizers, the pregnant mother exhibits a rhythm in various hormones, metabolites and body temperature that is transferred to the fetus. The fetus in turn develops in body movement, heart rate and hormone level rhythmicity in late gestation (left panel). Circadian disruption in the mother leads to adverse pregnancy outcomes in humans and has future health consequences in the offspring in animals (right panel). **(B)** During preterm birth, the infant loses temporal signals of the mother and is exposed to the NICU environment. Disruption of 24-h rhythmicity in the NICU may be associated with longer hospital stay and less weight gain in humans and might have long-term health consequences.

During pregnancy, the circadian rhythm of the fetus is primarily entrained by maternal cues. The mother rhythmically synthesizes hormones like melatonin, glucocorticoids, and neurotransmitters that pass the placenta, thereby transferring circadian signals to the fetus (Reiter et al., 2014a; Bates and Herzog, 2020). Not only hormonal cues, but also food intake, exercise, and body temperature may influence the fetal rhythm (Figure 1A). In summary, maternal signals function as Zeitgebers for the fetus throughout pregnancy. The human visual and circadian system gradually develops during the fetal and early postnatal period. Human eye development starts from week 4 of gestation, while the fetal SCN has been visualized by radioactive labeling from week 18 of gestation and shows characteristics of maturation from this time onwards (Reppert et al., 1988; Rivkees and Lachowicz, 1997). By midgestation, the SCN neurogenesis and innervation by the hypothalamic tract is complete. The photoreceptors that are required for non-image-forming irradiance detection contain the photopigment melanopsin and are likely the earliest to be functional in humans (Hattar et al., 2002). Studies in preterm baboons suggest that the human SCN may become light-responsive at 24 weeks of gestation, but evidence is limited (Hao and Rivkees, 1999; Hanita et al., 2009). Although the SCN’s metabolic rhythmicity has been detected at the end of pregnancy in primates (Seron-Ferre et al., 2012), clear evidence on the precise timing of the appearance of endogenous SCN rhythmicity in humans is still lacking. In mice, rhythmic expression of the first core clock components in the SCN has been detected around embryonic day 14, and intracellular synchrony between SCN cells increased as fetal development proceeded (Landgraf et al., 2014). In several peripheral tissues, rhythmic clock gene expression was detected around embryonic day 18–19 (Sladek et al., 2004; Dolatshad et al., 2010). In fetal rats, SCN rhythmicity in glucose utilization was detected 1–2 days before birth, but rhythmic clock gene and neuropeptide expression started to arise postnatally (Reppert and Schwartz, 1984). The fetal rat adrenal shows robust rhythms of *Per2* and *Bmal1* at E18, driving rhythmic secretion of corticosterone (Torres-Farfan et al., 2011). It remains to be investigated if the observed rhythmicity is driven by the maturation of the fetal SCN (i.e., increasing intrinsic rhythmicity) or by entrainment through (external) maternal cues.

Another distinction that is difficult to make is whether the maternal (endocrine) signals entrain the fetal SCN, or also directly target the fetal peripheral clocks. The neuropeptides vasopressin (AVP) and vasoactive intestinal polypeptide (VIP) are expressed in the SCN where they function as neuronal synchronizers and stimulate core clock gene expression (Ono et al., 2021). Swaab et al. (1990) have shown that AVP is detectable in the fetal hypothalamus from the 27th week of pregnancy but its rhythmic expression arises only after birth in humans. The VIP-producing neurons were first detected in week 31 of pregnancy and also start to show circadian rhythms postnatally. The neonatal SCN contains a small number of these AVP- and VIP-expressing neurons, indicating that this system matures further during the first years of life (Swaab et al., 1990; Swaab et al., 1994).

As mentioned previously, melatonin is synthesized in a circadian manner by the maternal pineal gland and is able to pass the placenta (Okatani et al., 1998; Seron-Ferre et al., 2012). At daytime, plasma levels of melatonin are low and increase during nighttime. It is thought that communication between the circadian system of the mother and the fetus predominantly goes via these differences in plasma melatonin levels. Interestingly, melatonin receptors are expressed on many fetal tissues (Williams et al., 1991). Animal research has shown that maternal pinealectomy early in gestation results in loss of normal temporal synchronization in drinking activity in the offspring, indicating that their SCN is n’t functional (Bellavia et al., 2006). When maternal melatonin injections were administered during late gestation, the effects of the pinealectomy on the drinking behaviour were reversed (Bellavia et al., 2006). In addition, another study showed that maintaining pregnant non-human primates under constant light conditions from 60% to 90% of gestation to suppress melatonin production caused altered clock gene expression in the fetal SCN (Torres-Farfan et al., 2006). The effect of melatonin suppression could be reversed by melatonin replacement. Taken together, this evidence suggests direct entrainment of the fetal SCN by the maternal endocrine system in rats and non-human primates, and that maternal melatonin is required for the development of the fetal circadian system.

Animal and human research has shown that the adrenal gland serves as a peripheral clock that receives signals from the SCN. As a result, glucocorticoids are produced in a circadian fashion (Oster et al., 2006). During gestation, the fetal adrenal gland is thought to respond to maternal endocrine signals. Torres-Farfan et al. (2006) showed that the temporal pattern of clock gene expression was identical between the fetal SCN and adrenal gland in capuchin monkeys. If the fetal adrenal clock was under control of the fetal SCN, a phase-delay between the SCN and the peripheral clocks would have occurred (Valenzuela et al., 2008). Furthermore, suppressing the maternal adrenal gland in humans using oral triamcinolone, a synthetic corticosteroid, led to disappearance of fetal rhythms in heart rate and limb movements (Arduini et al., 1986). We hypothezise that this may be due to the nonpyshiological levels of maternal cortisol and the subsequent disappearance of the 24-h cortisol rhythm (Koyanagi et al., 2006; Oster et al., 2017). This indicates that the cellular rhythms of the fetal SCN and peripheral clocks are probably driven by maternal signals rather than the molecular clock of the fetal SCN at this stage of development.

## Circadian rhythm and preterm birth

In the event of preterm birth, a tremendous mismatch with the uterine chronobiological environment arises (Figure 1B). The neonate loses rhythmic (hormonal) cues, normally received *in utero*, and is prematurely exposed to circadian synchronizers like daylight and enteral nutrition. The SCN and visual system have not completely matured, as this normally occurs throughout gestation and continues in the neonatal period after term birth (Swaab, 1995; Lammertink et al., 2020). Impaired maturation of the circadian system in preterm infants is likely a leading cause of delayed development of circadian rhythmicity after preterm birth (Rivkees and Hao, 2000; Rivkees, 2003). During the first month after birth, no clear diurnal patterns in activity, rest, or body temperature have been found (D'Souza et al., 1992; Anders et al., 1985; Glotzbach et al., 1995). A rhythm in temperature arises by 1 month of age, and after 6 weeks daytime sleep/wake cycles become more apparent (Kennaway et al., 1992). In line with this, day-night rhythms in hormone production become apparent, with diurnal production of melatonin detectable at 12 weeks of age (Kennaway et al., 1992). Melatonin does not only play a role in sleep-wake patterns but also exhibits anti-inflammatory, anti-carcinogenic and anti-oxidant functions (Reiter et al., 2014b). Interestingly, all these functions also show daily or circadian variations.

In preterm infants, studies on the timing of circadian rhythm emergence still show conflicting results. D'Souza et al. (1992) have shown that in the majority of the preterm infants born between 24–29 weeks of gestation no circadian rhythmicity in skin temperature and heart rate could be observed until they reached 34 weeks of gestation. Others have shown that an ultradian (i.e., shorter than 24-h) rhythm can be detected around 35 weeks gestational age, but no clear circadian rhythm could be detected. Guyer et al. (2012) have shown that very preterm infants admitted to the NICU show an earlier emergence of a 24-h sleep-wake rhythm compared to term infants at an equivalent age, indicating that exposure to environmental time cues plays a role in sleep-wake rhythm development. On the other hand, there is evidence suggesting that circadian sleep-wake patterns develop merely as a result of postnatal brain maturation, independent of environmental cues (Mirmiran et al., 2003). Further research is warranted to determine which factor is the major contributor to maturation of the circadian system in preterm infants.

As mentioned previously, circadian rhythms are entrained by environmental cues. After preterm birth, parental and nursing care in the NICU may influence the maturation of circadian rhythms. Physical contact (parental kangarooing) and nursing care affect heart rate, sleep, and stress levels of the infant (Santos et al., 2015); therefore it is likely that these factors drive, or at least stimulate, the observed ultradian rhythms. On the other hand, there are many factors that lack strict rhythmicity or disturb physiological rhythmicity in the NICU, including lack of a clear day/night rhythm in light and noise exposure, enteral and parenteral feeding schedules, stressful interventions (blood withdrawal, x-rays, etc.), physical contact, medication and sleep-wake rhythm disturbances. All these factors may alter the programming and maturation of organs and body functions such as development of the hypothalamic-pituitary-adrenal axis, the autonomic nervous system, and the circadian system itself (Lammertink et al., 2020).

## Early circadian disruption and later life disease

Epidemiological evidence in humans indicates that disruption of the circadian rhythm during pregnancy, caused by shiftwork, travel across time zones or exposure to light at night, can increase the risk of adverse birth outcomes such as miscarriage, preeclampsia and preterm delivery (Cai et al., 2019). Subsequently, long-term adverse health effects have been reported such as sleep disorders, cancer, susceptibility to infections, metabolic syndrome and aging (Van Dycke et al., 2015; Yu et al., 2015; Kecklund and Axelsson, 2016; Shimizu et al., 2016; Charrier et al., 2017; Cai et al., 2019; Logan and McClung, 2019; Longo et al., 2021). It is thought that these Non-Communicable Diseases (NCDs) arise because the crosstalk between the different physiological systems and the circadian system gets shifted, resulting in alterations in whole-body physiology. Although these findings imply that disruption of maternal and thereby fetal rhythms during pregnancy may hamper health and development, evidence on long-term effects in humans is still lacking.

Drawing parallels with the evidence for long-term health consequences of fetal circadian disruption during pregnancy, one may hypothesize that the same applies to circadian disruption of the preterm infant. Those born after very or extreme preterm birth (i.e., born before 28 weeks of gestation) spend the equivalent of their last trimester of fetal life in an unnatural environment. The developing preterm infant’s brain is highly sensitive to environmental exposures, which can cause alterations of neuronal networks and macroscopic brain structures including the hypothalamus (Lammertink et al., 2020). In accordance with the developmental origins of health and disease (DOHaD) theory, this can have life-long consequences. The DOHaD theory states that environmental exposures during the periconceptional period and in early life can lead to epigenetic and developmental adaptations, increasing vulnerability to disease in later life (Hanson and Gluckman, 2014).

The exact mechanism of how chronodisruption during pregnancy or shortly after birth may lead to disease predisposition in later life is unknown. It may partially be explained by the DOHaD theory, with epigenetic changes due to environmental influences. Circadian rhythm disruption and subsequent alterations in clock gene expression may be one of the underlying mechanisms leading to increased risk of neurodevelopmental as well as a variety of cardiometabolic diseases in human adults (Shimizu et al., 2016).

Animal studies provide evidence that maternal chronodisruption from the start of gestation using a frequently shifting light-dark schedule results in a pathological phenotype in the offspring (Chaves et al., 2019). Strinkingly, a similar pathological phenotype was observed in *Bmal1* deficient mice (Lefta et al., 2012). Increasing evidence has shown that timing of food intake is a potent synchronizer for the mammalian circadian system (Challet, 2013). This is controlled by the SCN and mediated through metabolic signals such as metabolites (glucose and fatty acids) and hormones (ghrelin, leptin, and insulin) (Moore and Eichler, 1972; Stephan and Zucker, 1972; Kalsbeek et al., 2011). Mistimed nutritional intake, for example, during the subjective night, leads to a transcriptional effect of the clock on metabolic pathways in peripheral organs such as liver, white adipose tissue, the adrenal gland, heart and kidney (Damiola et al., 2000; Storch et al., 2002; Turek et al., 2005; Zvonic et al., 2006; Hoogerwerf et al., 2007; Lamia et al., 2008). Circadian-related metabolic diseases after (gestational) chronodisruption are most likely caused by shifted rhythms in glucose, insulin, glucocorticoids, leptin and triglycerides leading to internal desynchronization (Panda, 2016). The endocrine changes caused by maternal chronodisruption can affect the fetal programming either directly or indirectly by altering uptake and delivery of nutrients by the placenta or fetal tissues (Fowden, 1995). In rats, alteration of the maternal circadian environment by exposure to chronic photoperiod shifting (CPS) causes impaired glucose tolerance and raises nocturnal blood pressure in the offspring (Mendez et al., 2016). Moreover, CPS exposed offspring showed increased accumulation of white adipose tissue as seen in obesity, hyperinsulinemia and low-grade inflammation (Varcoe et al., 2013; Leproult et al., 2014; Mendez et al., 2016). Since glucocorticoids are known to regulate fetal (circadian) development one may hypothezise that they play an important role in the emergence of these disturbances. Hence, evidence has shown that elevated maternal glucocorticoid levels can lead to hypertension, glucose intolerance and abnormal functioning of the HPA-axis in the offspring (Fowden, 1995; Bertram and Hanson, 2002). If these metabolic and hormonal disturbances are (partly) caused by a direct effect from glucocoids on fetal peripheral clocks remains to be investigated. In addition, Varcoe et al. (2016) showed that absence of maternal melatonin during pregnancy induces glucose intolerance in the offspring, suggesting that melatonin also plays a crucial role in the interplay between the circadian system and metabolic health. In humans, maternal night-time food intake has also been linked to impaired glucose tolerance and an increased risk of gestational diabetes mellitus and obesity (Colles et al., 2007). In addition, one study showed that in humans maternal shiftwork during pregnancy is associated with childhood overweight and metabolic disturbance in the offspring (Liao et al., 2022). It is important to note that a definite conclusion on the causality between these adverse health effects and circadian dysregulation cannot be drawn due to the complexity of these diseases and possible confounders that may be involved (such as sleep quality and quantity). In summary, these data indicate that maternal chronodisruption has far reaching consequences for the offspring due to alterations of whole-body physiology and pathologic changes in nearly all organ systems.

Subsequently, exposure to continuous bright light (CBL) or near darkness (ND) in the postnatal period can cause lasting alterations to the circadian system. In mice, it was found that exposing pups to CBL or ND conditions postnatally possibly leads to altered synchronization within SCN cell populations, impairing SCN responsiveness to light in the long term (Shimizu et al., 2016). Moreover, alterations in clock gene levels have been observed: the amplitude of Per2 rhythms in the SCN, heart, and lung of ND-reared mice was altered, while those in the liver were unchanged (Chaves et al., 2019). This might be because the liver’s main synchronizer is feeding time instead of light. Another research group has shown that postnatal exposure of rats to CBL leads to long-term alteration of SCN morphology and the animal’s metabolic state, resulting in a higher fat mass and loss of glucose and triglyceride rhythmicity (Madahi et al., 2018). Genetically, postnatal CBL exposure leads to a change in rhythmicity of most examined clock genes in the retina, SCN, and the pineal gland in these rats (Kubistova et al., 2020). Five out of seven examined genes were completely arrhythmic in the SCN at postnatal day 30 and one gene even at P90 (Kubistova et al., 2020). In terms of phenotype, mice and rats exposed to CBL postnatally have been shown to exhibit both anxiety- and depressive-like behavior (Borniger et al., 2014; Coleman et al., 2016). Collectively, these findings support the possibility that chronodisruption during the periconceptional period or early in life leads to misalignment of the timing of circadian and clock-controlled gene expression in multiple organ systems. This may cause long-term morphological, epigenetic and molecular alterations of whole-body physiology leading to an increased risk for NCDs. Further research on epigenetic changes due to early life circadian rhythm disruption and functional analysis of the consequences of altered clock gene expression should provide more insights into the mechanisms for disease predisposition.

## Rhythmic ques and current NICU guidelines

To date, little attention has been paid to rhythmic cues in NICUs. The only circadian synchronizers thus far taken into account within the NICU guidelines are the illumination and sound levels. In the past it has been suggested that since the womb is dark, preterm infants should be cared for in (near) dark conditions to promote growth, sleep, and (neuro) development (Als et al., 1994). In the Netherlands, incubator covers and dim lighting are therefore commonly used to provide semi-dark or dark conditions all day. Hellstrom-Westas et al. (2001) have shown that the use of these covers alters sleep patterns in the short-term, but the long-term effects are unknown. The NIDCAP (Newborn Individualized Developmental Care and Assessment Program) developed a method for “optimal” care for preterm infants in the NICU and has been shown to, among others, reduce days on mechanical ventilation and oxygen support, improved weight gain and shorter hospital stays (Als et al., 1994; Als et al., 2003). However, they do not address (cycled) light exposure in their approach. Although preclinical and clinical evidence suggests that reducing light exposure during the night leads to improved psychomotor development and sleep patterns, increased stability of the autonomic nervous system, faster weight gain, shorter NICU stay, and reduced stress in newborns, implementation in the NICU is difficult (Guyer et al., 2012; Vasquez-Ruiz et al., 2014; Moselhi Mater et al., 2019). This is most likely because well-designed randomized controlled trials are lacking and exposure to high-intensity lightning is known to increase stress and induce physiological changes (Ozawa et al., 2010).

At present, optimal NICU illumination is still under debate and differs per country and even per hospital. The most recent American guideline on NICU design recommended light exposure not to exceed 20 lux in preterm infants below 30 weeks of gestation (White and Consensus Committee on Recommended Design Standards for Advanced Neonatal Care, 2020). In the NICU, light levels may vary between 100–200 lux during the day and up to 50 lux during the night (White and Consensus Committee on Recommended Design Standards for Advanced Neonatal Care, 2020). These lux levels are recommended to minimize disruptions to infants’ sleep-wake patterns and to promote optimal growth and neurodevelopment. In addition, artificial lighting should be spectrally comparable to daylight and adjustable, since cycled light (CL) might be beneficial after 28 weeks of gestational age (White et al., 2013). Regarding acoustic characteristics, high noise levels in the NICU can lead to arousal, sleep disturbance, changes in brain activity, and hearing loss (Perlman, 2001; Kuhn et al., 2013; Wroblewska-Seniuk et al., 2017). In addition, evidence shows that noise exposure can alter clock gene expression in the SCN and inner ear (Gu et al., 2015; Fontana et al., 2019). General sources of noise in the NICU include the monitor alarms, incubator motor and closing of the incubators. The noise levels reached by these sources vary between 70–90 decibel (dB), far more than recommended, as the guideline mentioned above advises that combined continuous and transient sounds in any bed space should not exceed 45 dB (White and Consensus Committee on Recommended Design Standards for Advanced Neonatal Care, 2020). To our knowledge, the effects of cycling noise on circadian rhythmicity in the NICU have not been studied yet.

Other synchronizers in (neonatal) intensive care units of interest are (par)enteral feeding practices, caregiving, incubator temperature, phototherapy administration, sleep disturbance, timing of elective care procedures, and timing of providing medication. To our knowledge, there are currently no clinical recommendations for timing of exposure to these synchronizers. Results of the large Circadiem trial are expected to shed light on the effect of introducing a bundle of synchronized care, focused on light and noise exposure, and timing of medication in neonatal intensive care for very preterm infants (CIRCA DIEM Trial Information, 2000). However, what is not being addressed in that trial is supporting rhythmicity by administration of circadian time-matched human milk since variations in composition of nutritional intake may be more physiological (Seron-Ferre et al., 2012). Another gap in knowledge that needs to be addressed is the role of sleep during the neonatal preterm period, and specifically active sleep, which is crucial for early brain development. Respecting infant’s sleep cycles by individually adjusting care procedures may improve neurodevelopmental outcomes and decrease morbidities. Monitoring sleep stages bedside, to better synchronize nursing and stressful procedures, including regular care blood withdrawals, is promising (Sentner et al., 2022). Additionally, timing of administration of corticosteroids or neuro-active medication could be matched to physiological moments of action (i.e., mornings). However, scientific evidence for these suggestions is still lacking and future research is warranted. In the next two paragraphs we will elaborate on two important cues: light and feeding, their role in the development of circadian rhythms, and how improving their rhythmicity might lead to better clinical outcomes.

## Cycled light interventions in the NICU

Light is the strongest Zeitgeber for daily clock entrainment and has been suggested to positively influence the premature circadian system (Morag and Ohlsson, 2016). Various studies have, therefore investigated the effects of cycled light (CL) in the NICU versus continuous bright light (CBL) or near darkness (ND) conditions on short-term infant outcomes (Mann et al., 1986; Seiberth et al., 1994; Boo et al., 2002; Brandon et al., 2002; Mirmiran et al., 2003; Rivkees et al., 2004; Guyer et al., 2012; Watanabe et al., 2013; Vasquez-Ruiz et al., 2014; Kaneshi et al., 2016; Brandon et al., 2017; Lebel et al., 2017). All these studies had methodological challenges and limitations. They were not blinded due to the nature of the intervention, had small sample sizes, and only included short-term outcome measures. Additionally, the definitions of CBL or ND illumination and outcome measures differed between studies.

A Cochrane review from 2016 included nine studies conducted between 1986 and 2014 (Morag and Ohlsson, 2016). Three of them compared CL with CBL, with all outcomes in favor of the CL group (Mann et al., 1986; Miller et al., 1995; Vasquez-Ruiz et al., 2014) with shorter duration of hospitalization, higher increase in weight gain, shorter duration on mechanical ventilation and earlier start of oral feeding (Miller et al., 1995). Interpretation of the data however is impeded due to high heterogeneity in study designs limiting meta-analyses. Six other studies compared CL with ND, with meta-analyses being possible (Seiberth et al., 1994; Boo et al., 2002; Brandon et al., 2002; Rivkees et al., 2004; Guyer et al., 2012). These meta-analyses showed that the duration of hospitalization was significantly shorter when CL was started at 32 weeks [−12.7 days; 95% CI (−23 to −2.3), *n* = 77], but not when CL was started directly at birth [−4.67 days; 95% CI (−14.8 to 5.5) *n* = 170] (Morag and Ohlsson, 2016). Daily weight gain was not significantly different within all the included studies. A single study found no significant difference in days until start of oral feeding or days on mechanical ventilator (Brandon et al., 2002). The systematic review concluded that CL in preterm infants leads to a shorter hospital stay than both CBL and ND, although the quality of the evidence was assessed as low and several studies had small sample sizes.

Since the Cochrane review in 2016, four new studies on light-cycling in the NICU have been published. Two focused on CL versus CBL (Farahani et al., 2018; Moselhi Mater et al., 2019). Moselhi Mater et al. (2019) used eye covers versus “normal NICU lighting.” They showed that the use of eye covers at night reduced distress levels (*p* < 0.00) and enhanced quiet sleep, muscle tension and reduced crying (Moselhi Mater et al., 2019). The other study, comparing CL and CBL, found a non-significant decrease in hospitalization length and a significant increase in daily weight gain in the CL group (Farahani et al., 2018). These findings show similar trends to the previously mentioned studies. Additionally, one study compared CL to ND (Chaves et al., 2019). The authors found no significant differences in physiological stability measures like heart and respiratory rate, suggesting that CL conditions do not lead to adverse events (Chaves et al., 2019). Finally, another study compared early (28 weeks gestational age) and late (36 weeks gestational age) introduction of CL and found non-significant improvements in weight gain and hospital stay in the early CL group (Brandon et al., 2017).

Furthermore, two Japanese studies have investigated practical solutions for creating CL conditions in the NICU, utilizing the detection spectrum of melanopsin (Watanabe et al., 2013; Kaneshi et al., 2016). Watanabe et al. (2013) covered NICU incubators with red light filters that block the wavelengths detectable by the infant’s melanopsin photoreceptors during the night. Consequently, the infants remain visible for caregivers while the synchronizing effect through the immature retina is blocked. When comparing infants in the light filter group to CBL they found a significant increase in day-night activity ratio at 38 weeks, but not at 34 weeks gestational age (Watanabe et al., 2013). The increase in weight gain at 60 weeks gestational age was significantly higher in the red filter group (intervention). Kaneshi et al. (2016) investigated whether using a red light, undetectable by melanopsin photoreceptors, during nursing at night would show improvement in comparison to the use of white light. They found no significant differences in activity patterns, night-time crying and weight gain, suggesting that short light exposure of infants during the night does not disturb their circadian rhythm development (Kaneshi et al., 2016). Combining these findings, CL conditions in the NICU could either be achieved through red light filters or by dimming the lights at night except for during nursing. In addition, another possibility would be the removal of the incubator cover during the day. However, more research on the effects of the infant’s distress levels is required prior to implementation in clinical setting.

Something to take into account regarding the implementation of cycled light conditions in the NICU is that approximately 80% of the preterm infants suffers from jaundice and are treated with intensive phototherapy (Rennie et al., 2010). The required duration of phototherapy depends on the severity of jaundice and should be administered until a statisfactory decline in serum bilirubin level occurs, which can vary from less than 24 h to several days (American Academy of Pediatrics Subcommittee on Hyperbilirubinemia, 2004). During treatment, only short breaks up to 30 min are recommended. To the best of our knowledge, time-of-day is currently not taken into account when administering phototherapy. Regarding the effect of phototherapy on the circadian rhythm, Chen et al. (2005) showed that blue light phototherapy in full-term infants altered the expression of circadian genes *BMAL1* and *CRY1* and plasma melatonin levels in peripheral blood mononuclear cells (PBMC), indicating that phototherapy affects the circadian system. Interestingly, they state that this response is mediated completely via extraocular light exposure since the eyes were covered with black cloth. This raises the question whether eye covers block the blue light signal and detection by the retina completely, since the role of extraocular light in circadian physiology is still questionable (Eastman et al., 2000; Ruger et al., 2003). In addition, the participants in the study of Chen et al. (2005) were all full-term infants, while in the NICU the majority is very preterm. Therefore, the results might not be fully translatable. Nevertheless, a retrospective cohort study conducted by van der Linden et al. (2023) demonstrated that phototherapy has a significant impact on 24-h light-dark cycles in preterm infants in the NICU. Although the same eye covers were used, this suggests that phototherapy does affect the circadian sytem in preterm infants. Future studies are required to gain insight in the short- and long-term consequences of intensive phototherapy on the development of the circadian system, including further elucidation of the underlying mechanism of action. In addition, we need to explore possibilities for future light interventions in the NICU while taking the circadian effects from phototherapy into account.

## Chrono-nutrition in the NICU

A circadian cue which to date has not been clinically investigated in preterm infants is the use of chrono-nutrition. McKenna and Reiss suggested a chrono-lactomics approach to feeding and breast milk composition for preterm infants in the NICU to improve postnatal (circadian) development (McKenna and Reiss, 2018). Very preterm infants are fed parenterally (continuously) and enterally after birth. Although no human or animal studies have been performed on this topic, the continuous intravenous provision of carbohydrates, proteins and lipids, independent of time of the day, may disturb the circadian rhythmicity of metabolic processes, which may negatively affect the maturation of the circadian system of preterm infants. It is important to note that during pregnancy, the placenta plays an important role in maternal-fetal nutrient transport via a complex interplay between placental transporters, maternal hormones, oxygenation, and nutrient concentrations (Jones et al., 2007).

In a hospital setting, neonatal enteral feeding (human milk or formula feeding) is provided on a regular basis (depending on birth weight and/or gestational/postnatal age, e.g., every 2 hours), without variation in quantity or timing over the day. Recently, evidence has shown that human milk can be regarded as a Zeitgeber since human milk composition varies throughout the day (Hahn-Holbrook et al., 2019). These circadian variations are thought to transfer important time-of-day information from the mother to the infant (Hahn-Holbrook et al., 2019). During the daytime, human milk contains components that promote activity such as neuroactive amino acids, immunological signals and cortisol, while at night, melatonin and tryptophan levels rise and total fat content increases (Guthrie et al., 1977; Illnerova et al., 1993; Cubero et al., 2005; Kent et al., 2006). Cubero et al. (2006) have demonstrated that infants fed with dissociated day or night formula milk showed improvement in all nocturnal sleep parameters such as total sleep, sleep efficiency and nocturnal awakenings, indicating that milk composition plays an important role in the development of the circadian system. In addition, other studies have shown that breastfed infants develop circadian rhythmicity in body temperature significantly earlier compared to formula-fed infants (Lodemore et al., 1992), suggesting that human milk promotes the development of the circadian system. Furthermore, Booker et al. (2022) have shown preliminary evidence that mistimed breast milk affects sleep onset and nighttime awakenings in infants born at term. Since the circadian system of preterm infants is not fully matured yet, synchronization of composition and provision of expressed human milk to the infant may facilitate the maturation and synchronization of the neonate’s circadian system. In contrast, unsynchronized milk may disrupt or delay circadian development. In order for the milk to be fully synchronized, the circadian rhythm of the mother has to be optimal (i.e., no shiftwork or jetlag). Whether synchronization of milk is feasible in the NICU setting and has beneficial effects on the infant’s sleep homeostasis and short- and long-term health, needs further study.

As these theories potentially have major impact on health and development of preterm infants, more research is required on optimizing parenteral and enteral nutrition in the NICU. We hypothesize that optimization of the mother’s circadian rhythm, an optimized feeding strategy and circadian-matched provision of milk may improve circadian development and simultaneously promote the neonate’s growth and health while minimizing the long-term risk of chronic diseases.

## Conclusion and future perspectives

In summary, the presented evidence underlines the importance of entrainment of the circadian rhythm in preterm infants. Although many questions remain unanswered, there is increasing evidence that caring for preterm infants in cycling light conditions is not harmful and has beneficial short-term effects. Furthermore, animal studies show that there might be long-term health consequences of circadian rhythm disruption in early life. Studies with larger sample sizes and longer follow-up are necessary to unravel other effects of CL conditions on preterm infants’ health. This knowledge could be achieved through randomized controlled trials or via cohort studies comparing CL and previous (CBL or ND) practice, since the new guidelines mention that cycling light in NICUs may be beneficial (White et al., 2013). Additionally, other circadian synchronizers like feeding- and medication timing, noise levels and temperature variations should be investigated further. Especially since the visual system is not fully developed in preterm infants and they would have received non-visual temporal signals *in utero*. Although the infants are bound to prescribed feeding schedules, 24-h rhythmicity might be stimulated by oscillations in nutritional caloric load or using circadian-matched (human) milk at a specific time of the day. Finally, mechanistic insights into the long-term effects of circadian clock programming are necessary. Since weight gain was one of the observed short-term effects, metabolic disease might be a logical starting point. Animal studies on the epigenetic effects of perinatal CBL or ND exposure could provide insights, since epigenetic alterations in early life are known to be associated with later life disease. Furthermore, the known alterations in gene expression due to early life circadian disruption should be investigated functionally in animal and *in vitro* models in order to access their influence on organ and cell function and provide a possible link to (metabolic) diseases.
